# A synthetic switch based on orange carotenoid protein to control blue–green light responses in chloroplasts

**DOI:** 10.1093/plphys/kiac122

**Published:** 2022-03-15

**Authors:** Luca Piccinini, Sergio Iacopino, Stefano Cazzaniga, Matteo Ballottari, Beatrice Giuntoli, Francesco Licausi

**Affiliations:** 1 Plantlab, Institute of Life Sciences, Scuola Superiore Sant’Anna, Pisa 56127, Italy; 2 Department of Plant Sciences, University of Oxford, Oxford OX1 3RB, UK; 3 Department of Biotechnology, University of Verona, Verona 37134, Italy; 4 Department of Biology, University of Pisa, Pisa 56126, Italy

## Abstract

Synthetic biology approaches to engineer light-responsive systems are widely used, but their applications in plants are still limited due to the interference with endogenous photoreceptors and the intrinsic requirement of light for photosynthesis. Cyanobacteria possess a family of soluble carotenoid-associated proteins named orange carotenoid proteins (OCPs) that, when activated by blue–green light, undergo a reversible conformational change that enables the photoprotection mechanism that occurs on the phycobilisome. Exploiting this system, we developed a chloroplast-localized synthetic photoswitch based on a protein complementation assay where two nanoluciferase fragments were fused to separate polypeptides corresponding to the OCP2 domains. Since Arabidopsis (*Arabidopsis thaliana*) does not possess the prosthetic group needed for the assembly of the OCP2 complex, we first implemented the carotenoid biosynthetic pathway with a bacterial β-carotene ketolase enzyme (crtW) to generate keto-carotenoid-producing plants. The photoswitch was tested and characterized in Arabidopsis protoplasts and stably transformed plants with experiments aimed to uncover its regulation by a range of light intensities, wavelengths, and its conversion dynamics. Finally, we applied the OCP-based photoswitch to control transcriptional responses in chloroplasts in response to green light illumination by fusing the two OCP fragments with the plastidial SIGMA FACTOR 2 and bacteriophage T4 anti-sigma factor AsiA. This pioneering study establishes the basis for future implementation of plastid optogenetics to regulate organelle responses upon exposure to specific light spectra.

## Teaser

Inspired by the light-driven conformational transitions of orange carotenoid proteins of cyanobacteria, a molecular device can switch its dimeric state in response to blue–green light.

## Introduction

Synthetic biology research applies engineering principles to a multidisciplinary approach to decompose natural systems into their essential components and reassemble them to produce novel functions (Gutmann, 2011). Among the numerous strands of synthetic biology, a particularly prolific area of research concerns the engineering of photoreactive proteins with signaling potential, such as photoreceptors (Olson and Tabor, 2014). Indeed, light quality and quantity, and their dynamics, provides valuable information for cells to integrate with other exogenous and endogenous cues and respond accordingly. Several chimeric constructs have been developed so far, with a focus on the control of neuronal activity by light, thus establishing the field of optogenetics (‘Method of the Year 2010’, 2011).

Light is essential for photosynthetic organisms and thus impacts most of their physiological processes, including development, growth polarity or movement, the internal clock(s), and interaction with the biotic and abiotic environment (Jiao et al., 2007). Naturally occurring photoreceptors typically consist of a prosthetic chromophore and an apoprotein, responsible for light perception and signal transduction, respectively. This generic description applies to most of the photoreceptors found in plants, including tetrapyrrole-binding phytochromes, flavin-based cryptochromes, and receptors that contain Light–Oxygen–Voltage motifs and rhodopsins (Sineshchekov et al., 2002; Paik and Huq, 2019). An exception is instead represented by UVB-RESISTANCE 8 (UVR8) and homologs, whose UV light-sensing ability is intrinsic to the protein, by virtue of two to four tryptophan residues (Tilbrook et al., 2013). Each photoreceptor perceives a specific range of the visible light spectrum, with some overlaps (Paik and Huq, 2019). Their sensitivity concentrates over red and blue wavelengths, which are especially relevant for photosynthesis, and the violet-blue wavelengths, for their high energy and thus potential harm to cells. In response to these stimuli, angiosperms photoreceptors control pigments biosynthesis, clock entrainment, phototropism, reproductive development (flowering and tuberization), stomatal opening, and chloroplast movement (Möglich et al., 2010).

To the repertoire of naturally occurring photoreceptors, synthetic biologists have developed additional ones, exploiting the possibility of domain shuffling across organisms. This has expanded the possibility to fine-tune molecular processes in response to specific light stimuli. Several plant photoreceptors and their protein partners have been deployed to harness light-elicited processes in animal cells, for example, through engineering *Arabidopsis thaliana* FLAVIN-BINDING, KELCH REPEAT, F BOX 1 (Yazawa et al., 2009), cryptochromes (Kennedy et al., 2010), UVR8 (Chen et al., 2013), and phytochromes (Müller et al., 2013). On the other hand, plant optogenetics has lagged behind, due to the extent overlap with a plethora of endogenous photoreceptors. Moreover, the intrinsic need of plants for light to grow poses an intrinsic experimental limitation to optogenetics applications. Pioneering efforts in this direction include protein mutagenesis aimed at producing slow-photocycling phototropin variants (Hart et al., 2019), engineering green light perception through synthetic transcriptional regulators inspired to prokaryotic systems (Chatelle et al., 2018), dual control of gene expression systems with monochromatic light while keeping the system in the off state during plant growth (Ochoa-Fernandez et al., 2020) and rhodopsin-mediated regulation of membrane potential (Zhou et al., 2021). Main approaches to the engineering of synthetic genetic switches in plants have been recently reviewed (Andres et al., 2019).

Few studies so far sought to engineer additional layers of regulation in plant organelles, such as mitochondria and chloroplasts, through synthetic biology approaches, to improve the efficiency of cellular energy factories (Nielsen et al., 2013; Xiang et al., 2020), or to introduce novel methods of transcriptional regulation (Verhounig et al., 2010). Here we propose the implementation of a chloroplast-localized synthetic photoswitch, based on a photoreceptor-associated protein complementation assay (PCA). PCAs exploit the affinity between two peptides fused to split-protein fragments that, when brought in proximity, reconstitute the activity of the original protein (Merezhko et al., 2020; Wang et al., 2020).

Our photoswitch system is based on the cyanobacterial orange carotenoid proteins (OCPs) (Kay Holt and Krogmann, 1981; Kerfeld et al., 2003), which, when activated by blue–green light, undergo reversible conformational changes that enable phycobilisome photoprotection (Wilson et al., 2006, 2008; Kirilovsky and Kerfeld, 2013). OCPs are water-soluble proteins, whose structure is mainly composed of a C-terminal domain (CTD) and an N-terminal domain (NTD) connected by a flexible linker (Wu and Krogmann, 1997; Kerfeld et al., 2003). The OCP apoprotein noncovalently incorporates a single keto-carotenoid molecule, such as canthaxanthin, 3′-hydroxyl-echinenone, or echinenone, as prosthetic group buried inside the two domains (Punginelli et al., 2009; Yaroshevich et al., 2021). OCP photoactivation is accompanied by extensive reconfiguration of carotenoid–protein interactions, with the consequent translocation of the pigment within the protein (Gupta et al., 2015; Leverenz et al., 2015; Bondanza et al., 2020). Photoconversion is possible only when a keto-carotenoid is buried inside the protein, whilst association with other carotenoids inhibits its photoconversion ability (Punginelli et al., 2009; Wilson et al., 2011). The photoconversion of OCP proteins has been exploited in the past to engineer energy transfer between molecules (Andreoni et al., 2017) and fused to fluorescent proteins to generate a nano-sized temperature sensor (Maksimov et al., 2019). Moreover, it was shown that separated NTD and CTD of OCPs can reversibly interact in a light-dependent manner in the presence of a keto-carotenoid (Lechno-Yossef et al., 2017; Moldenhauer et al., 2017).

Here, we report the genetic engineering of Arabidopsis plants to express a chimeric photoswitch based on the *Fischerella thermalis* OCP2 and the NanoLuc luciferase protein (England et al., 2016), together with a bacterial enzyme for keto-carotenoid biosynthesis to generate the necessary prosthetic group (Ruiz-Sola and Rodríguez-Concepción, 2012; Nisar et al., 2015; Bai et al., 2017). We describe the application of transient and stable transformation systems to fully characterize the dynamics of such synthetic construct and the application of the OCP2-based photoswitch to control gene expression in chloroplasts.

## Results

### Design of a photo-switchable device based on OCP properties

**Figure 1:**
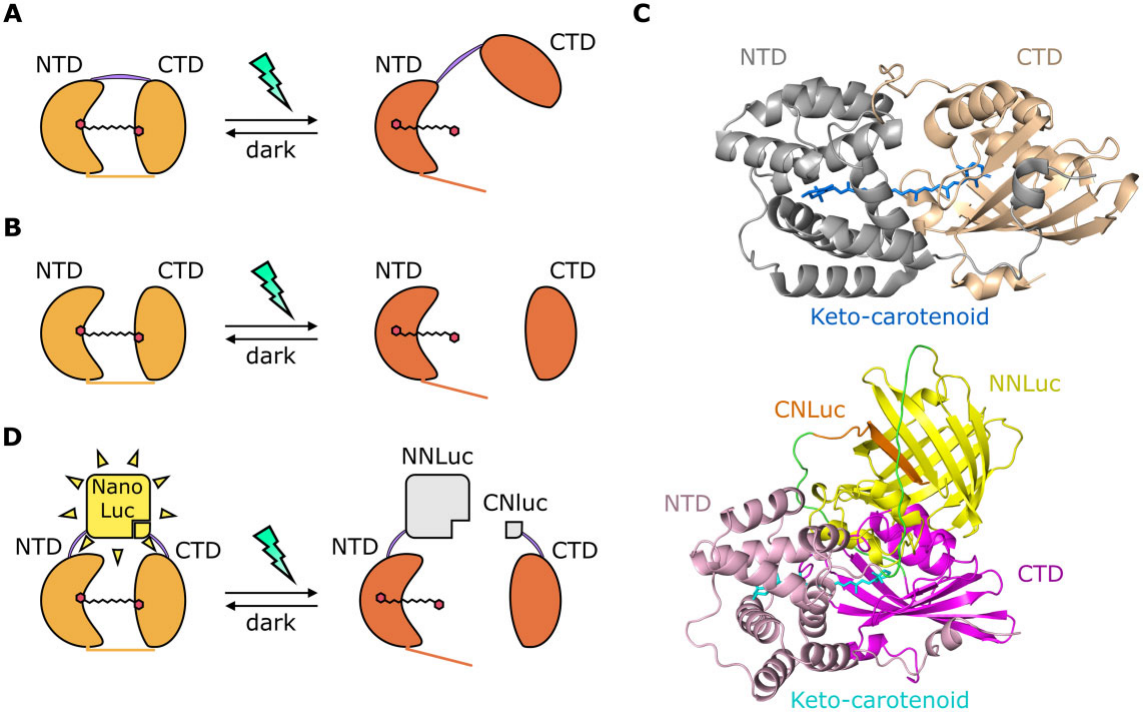
Rational design of a blue–green photoswitch based on OCP2 modules and the NanoLuc complementation system (split-NLucOCP2). A, Scheme of the light-induced native OCP2 protein dynamics: inactive state (left), called OCP2 orange (OCP2^°^), and light-induced active state (right), known as OCP2 red (OCP2^R^). The purple line indicates the flexible linker connecting the two domains, the orange line indicates the N-terminal extension. B, NTD and CTD interactions, when expressed as separate peptides, in darkness (left) or under stimulating light. C, In silico prediction of dark OCP2 (top) and dark split-NLucOCP2 (bottom) 3D structures. D, Scheme of the split-NLucOCP2 photoswitch. In the dark, the two OCP2 modules interact with each other, thus bringing the two NanoLuc fragments in proximity and complementing NanoLuc; blue–green light leads to a separation of the two modules, suppressing NanoLuc activity.

To engineer a light-dependent molecular switch to be orthogonally active in plant plastids, we focused on the class of the cyanobacterial OCPs. Among the three known paralogous families of OCPs (OCP1, OCP2, and OCPX; Melnicki et al., 2016; Bao et al., 2017), we reckoned the OCP2 family to fit better the rational design of a synthetic photoreceptor. Indeed, its monomeric state and its ability to revert to the dark-adapted state (OCP2^°^) with faster kinetics than the OCP1 in the absence of a helper protein (fluorescence recovery protein; Bao et al., 2017) reduce the number of components required for the photoswitch to function. Moreover, in NTD–CTDs heterodimerization assays, only *Fremyella* OCP2 domains reverted to the dark state after photoconversion, while this was not observed for OCP1 (Lechno-Yossef et al., 2017). Thus, we selected the OCP2 coding sequence from the cyanobacterium *F.* *thermalis*, hereafter *F. thermalis*, and separated its NTD (165 amino acid [aa]) and CTD (131 aa), getting rid of the flexible linker between them. *Fremyella* *thermalis* was chosen as the source organism as one of the few known to encode for an OCP2 protein as the sole OCP sequence in its genome (Bao et al., 2017). Moreover, being *F. thermalis*, a thermophilic organism (Alcorta et al., 2019), proteins encoded by its genome likely have higher stability compared to the case of nonthermophilic ones. To test whether these modules reconstitute a complex capable of light-dependent regulation in angiosperm cells, we generated a synthetic construct that couples a luminescent output with dimerization (Figure 1). In our design, we exploited the affinity and light-driven separation of the two OCP2 modules for a PCA, based on a NanoLuc complementation assay developed in mammalian cells and called NanoBit (Dixon et al., 2016).

We fused each domain with one of the two fragments of the split-NanoLuc protein: the N-terminal (NNLuc, 159 aa) and the C-terminal (CNLuc, 11 aa) fragments. In this way, we generated two chimeric constructs which, together, constitute a split-NLucOCP2 photoswitch. In the wild-type OCP2 protein, the two domains associate by means of a flexible linker and by the noncovalent bonds that a single keto-carotenoid molecule establishes after its incorporation inside the OCP2 protein (Wu and Krogmann, 1997; Kerfeld et al., 2003; Figure 1A). Instead, in our synthetic construct, the OCP2 modules are kept together exclusively by their interactions with the keto-carotenoid (Figure 1B). Since the strength of these interactions depends on the light-dependent translocation of the keto-carotenoid inside the OCP2 structure (Gupta et al., 2015; Leverenz et al., 2015; Bondanza et al., 2020; Kuznetsova et al., 2020), we expected the NTD and the CTD to associate in the dark, whereas exposure to blue–green light would bring them apart.

Specifically, we fused the NNLuc to the C-terminus of the NTD module (NTD-NNLuc), and the CNLuc to the N-terminus of the CTD module (CNLuc-CTD). This conformation was selected following an in silico prediction based on the 3D structures of NanoLuc (Protein Data Bank [PDB] ID 5IBO) and the OCP from *Arthrospira maxima* (PBD ID 5UI2), which shares the strongest sequence similarity with FtOCP2 among the OCPs whose structure has been experimentally determined, since no OCP2 structure has been resolved yet (Supplemental Figure S1). This model suggested these fusions as the best option to bring the NanoLuc modules in close proximity in the closed (OCP^°^) conformation (Figure 1C).

We predicted that the interaction between the NTD and the CTD in the dark would reconstitute the enzymatic NanoLuc activity and therefore produce a luminescent output upon addition of the furimazine substrate to plant cell extracts (Hall et al., 2012; Dixon et al., 2016). On the other hand, we expected blue–green light exposure to promote detachment of the CTD from the NTD, resulting in the disruption of the NanoLuc complementation (Figure 1D). The low affinity between the NNLuc and the CNLuc themselves (Dixon et al., 2016), allowed us to rule out that the reconstitution of the NanoLuc activity would only be due to their interaction, while it is, instead, the result of the affinity between the two OCP2 modules.

### Generation and characterization of canthaxanthin-producing Arabidopsis transgenic plants

**Figure 2:**
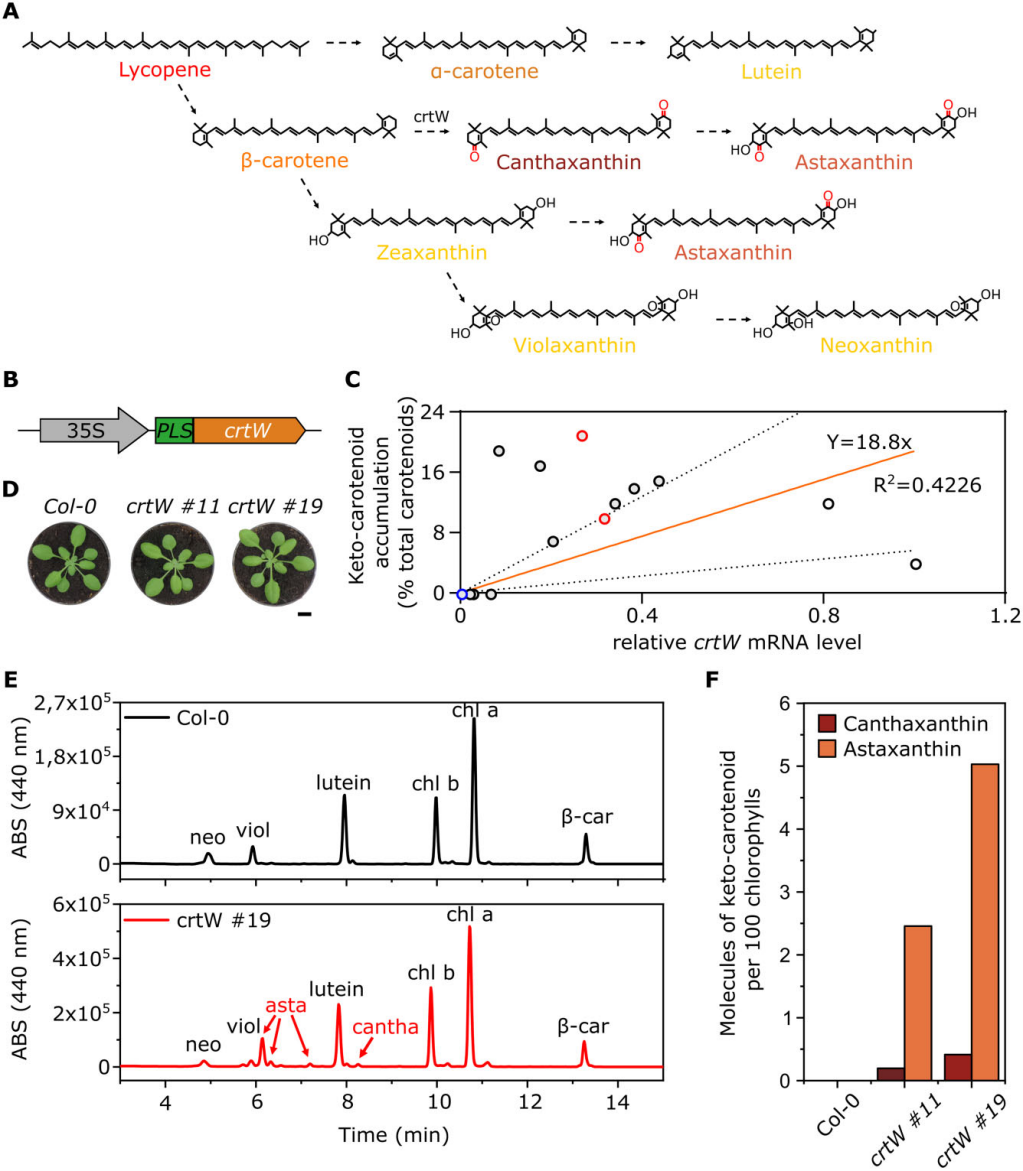
Pigment content analysis of keto-carotenoid *A. thaliana* transgenic lines. A, Overview of the biosynthetic carotenoid pathway after the addition of the crtW enzyme; dotted arrows represent multiple enzymatic steps. B, Scheme of the synthetic construct for canthaxanthin production: CaMV 35s promoter; PLS; β-carotene ketolase (crtW). C, Relative *crtW* mRNA levels and their corresponding keto-carotenoid content in 13 independent transgenic lines (black and red circles) and one Col-0 (blue circle); red circles represent the two lines (#11 and #19) chosen for the following experiments. mRNA levels in the graph are relative to the transgenic line with the highest expression, set as 1. The orange line represents the curve fitted to the data with linear regression, and the dotted lines are the 95% prediction confidence intervals. The *R*^2^ and the equation of the curve are shown. D, Phenotypic comparison between Arabidopsis Col-0 plants and two independent *crtW* transgenic lines. The images in (D) were digitally extracted for comparison. Scalebar 1 cm. E, HPLC analysis for canthaxanthin and astaxanthin detection in a *crtW* line (red line), compared with a Col-0 plant (black line); neoxanthin (neo), violaxanthin (viol), astaxanthin (asta), canthaxanthin (cantha), chlorophyll b (chl b), chlorophyll a (chl a), β-carotene (β-car). F, Quantitative measurement of canthaxanthin and astaxanthin from isolated thylakoids (one single extract from 20 3-week-old, ketocarotenoid-producing plants), represented as molecules of keto-carotenoid per 100 molecules of chlorophylls.

Both the interaction and the photoconversion of the OCP2 modules depend on the presence of specific keto-carotenoids (canthaxanthin or echinenone; Punginelli et al., 2009; Kuznetsova et al., 2020). Therefore, we generated stable transgenic Arabidopsis lines expressing the β-carotene ketolase (crtW) enzyme from *Agrobacterium aurantiacum*, which catalyzes canthaxanthin biosynthesis from β-carotene (Figure 2A), and astaxanthin biosynthesis from zeaxanthin, via the addition of a carbonyl group to carbon 4 and 4′ of the substrate (Misawa et al., 1995; Choi et al., 2005; Bai et al., 2017). Moreover, canthaxanthin itself can be hydroxylated by endogenous hydroxylase enzymes, leading to astaxanthin production (Zhong et al., 2011). Since carotenoid biosynthesis in angiosperms occurs in plastids (Nisar et al., 2015), we fused the sequence of the crtW enzyme with a previously tested plastid localization sequence (PLS) from ribulose 1,5 bisphosphate carboxylase 3 (RBCS-3) of pea (*Pisum sativum*), and placed this transgene under the control of the constitutive *Cauliflower mosaic virus* (CaMV) 35S promoter (Figure 2B).

Following positive selection on kanamycin, 21 putative transformants were isolated. To evaluate the actual expression of the transgene, we measured the *PLS-crtW* transcript level through reverse transcription-quantitative PCR (RT-qPCR) and correlated it to the content of keto-carotenoids, as measured by high performance liquid chromatography (HPLC; Figure 2C). Thirteen *35S:PLS-crtW* plants actively expressed the transgene with extensive variability. Out of these 13 lines subsequently analyzed, 10 produced detectable levels of keto-carotenoids, ranging from 4% to 21% of the total carotenoid content (Figure 2C). Astaxanthin was the most abundant keto-carotenoid accumulated, but all 10 lines also contained canthaxanthin.

Two independent lines with high and intermediate keto-carotenoid content (Figure 2C, red circles, and Figure 2D) were further analyzed by isolation and quantification of the keto-carotenoid content in the thylakoids (Supplemental Table S1; Figure 2, E and F). Keto-carotenoids were synthesized at the expense of the β–β xanthophyll neoxanthin and violaxanthin, which were reduced with respect to the parental lines. Zeaxanthin was not detected in any plant in the light regime used in this analysis. The two transgenic lines showed a slightly brown pigmentation compared to the wild-type, which was confirmed by a phenotyping analysis based on red, green, and blue (RGB) imaging of the plants through a commercial phenotyping machine. Comparing Columbia-0 (Col-0), *crtW #11*, and *crtW #19* genotypes at 17, 21, 24, and 28 d after germination, we found no significant difference in the average size of the plants (Supplemental Figure S2A), but a comparison of the HUE-value (derived from the three components of the RGB images) showed a statistically significant decrease in both the *crtW* lines compared to the Col-0 (Supplemental Figure S2B). Considering the gathered evidence collectively, we concluded that the implementation of the endogenous carotenoid biosynthetic pathway allowed the production of keto-carotenoids in Arabidopsis.

### Testing of OCP2-based photoswitch in plant protoplasts

We generated plasmids carrying the NTD-NNLuc and CNLuc-CTD transgenes, each equipped with a PLS, to be expressed under control of the 35S CaMV promoter, and we tested the functionality of the photoswitch system in transient assays in mesophyll protoplasts. We started by investigating the activity of the constructs in isolated protoplasts from either Col-0 or *crtW* plants, to assess whether keto-carotenoids were required for the output of the system, according to our rational design.

**Figure 3:**
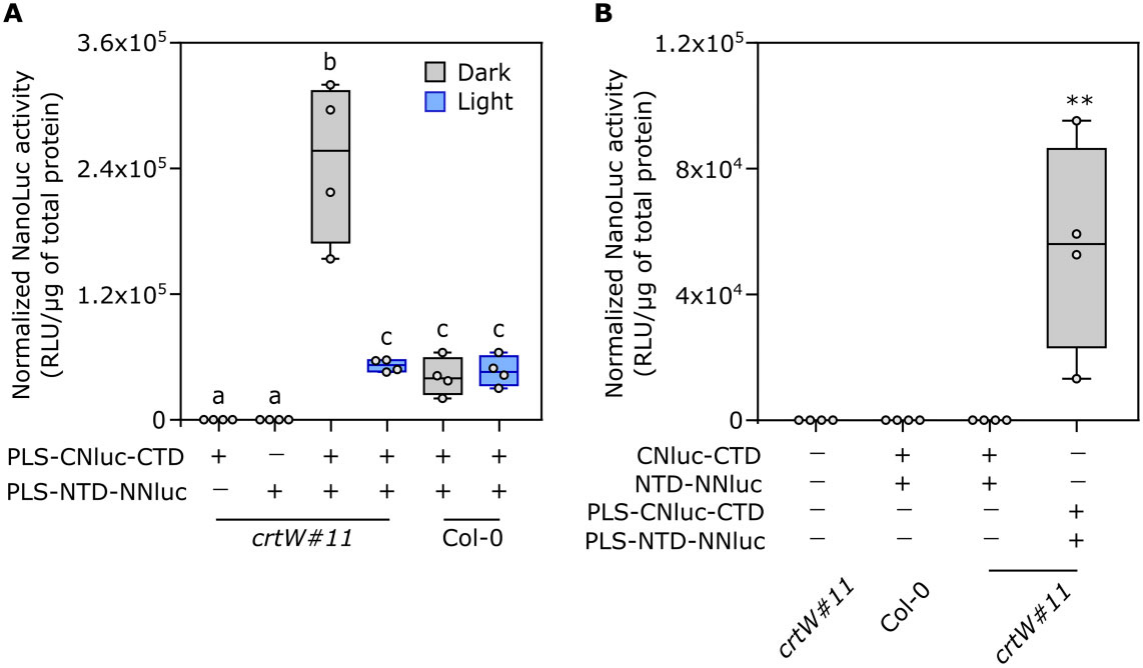
Activity of the synthetic photoswitch in a transient protoplasts assay. A, Light response in Arabidopsis Col-0 and transgenic *crtW*-isolated protoplasts. Different letters indicate statistical differences (*P*  ≤  0.05) calculated from one-way ANOVA (*n* = 4) followed by Tukey’s post-hoc test. “Light” indicates treatment with under blue light (465–480 nm, 350 µmol μm^−2^ s^−1^) for 1 h. B, Comparison between plastidial and nonplastidial localization of photoswitch components. Protoplasts were treated with either dark or blue light (350 µmol μm^−2^ s^−1^) for 1 h. Asterisks indicate statistical differences calculated from one-way ANOVA (*P* ≤ 0.01, *n* = 4). In the box plots, dots represent biological replicates, the black line marks the median, and the box indicates the interquartile range (IQR). Whiskers extend to data points below 1.5× IQR away from the box extremities.

A first proof-of-principle experiment was performed to evaluate the responsivity of the system to the blue–green light stimulus and its basal activity. All possible combinations (Figure 3A) of the two modules were transformed in *crtW* protoplasts. Sixteen hours after the transformation, dark-incubated protoplasts were treated under blue light (465–480 nm, 350 μmol m^−2^ s^−1^) for 1 h, while control samples were maintained in the dark. Protoplasts transformed with CNLuc-CTD or NTD-NNLuc alone showed no luminescent output in the dark, confirming that the two NanoLuc fragments did not retain any basal enzyme activity (Figure 3A). Those transformed with both modules were instead analyzed for light response. High luminescence output in dark-treated protoplast was suggestive of effective complementation and reconstitution of a dark-adapted state in the OCP2 complex. The output was instead significantly lower in illuminated protoplasts, indicating the detachment of the OCP2 modules, as expected, with consequent disruption of the enzymatic activity (Figure 3A). Wild-type protoplasts had identical output upon dark or light treatment, suggesting that light is unable to trigger the structural changes in the OCP2 complex in the absence of the proper keto-carotenoids. The residual activity recorded in the wild-type background pointed at the occurrence of some extent of unspecific interaction between the two photoswitch modules, likely due to the affinity of the OCP2 fragments. Remarkably, however, blue-light treatment in the *crtW* background cut the output down to the level associated with unspecific module interaction (Figure 3A), suggesting that the keto-carotenoid-dependent changes that are typical of the native OCP2 protein were fully recapitulated in our split configuration.

Next, we investigated the effect of a different subcellular localization of the photoswitch modules. In plants, carotenoids are mainly concentrated inside plastids, and no specific or generic carotenoid export system is known (Sun et al., 2018). To exclude our photoswitch from plastids, we generated two new versions of the CNLuc-CTD and NTD-NNLuc modules devoid of PLS. The new modules were again tested both in *crtW* and Col-0 protoplasts, using the plastid localized photoswitch as positive control for the NanoLuc activity. We found that the luminescent signal produced in the cytosol-localized photoswitch modules was comparable to that of untransformed protoplasts (Figure 3B), confirming that our photoswitch can only be active in carotenoid-containing organelles. To assess whether the OCP-switch could benefit from higher keto-carotenoid levels or these actually saturated OCP2 occupancy, we repeated the experiment shown in Figure 3A, comparing protoplasts isolated from the *crtW* line #11 with those from #19, which showed different keto-carotenoid content (Figure 2F). The decrease of Nanoluc activity after light treatment was comparable in the two independent lines (Supplemental Figure S3), indicating that keto-carotenoid concentrations saturated OCP complexes in both transgenic lines.

### Characterization of split-NLucOCP2 light responsiveness and reversion

**Figure 4:**
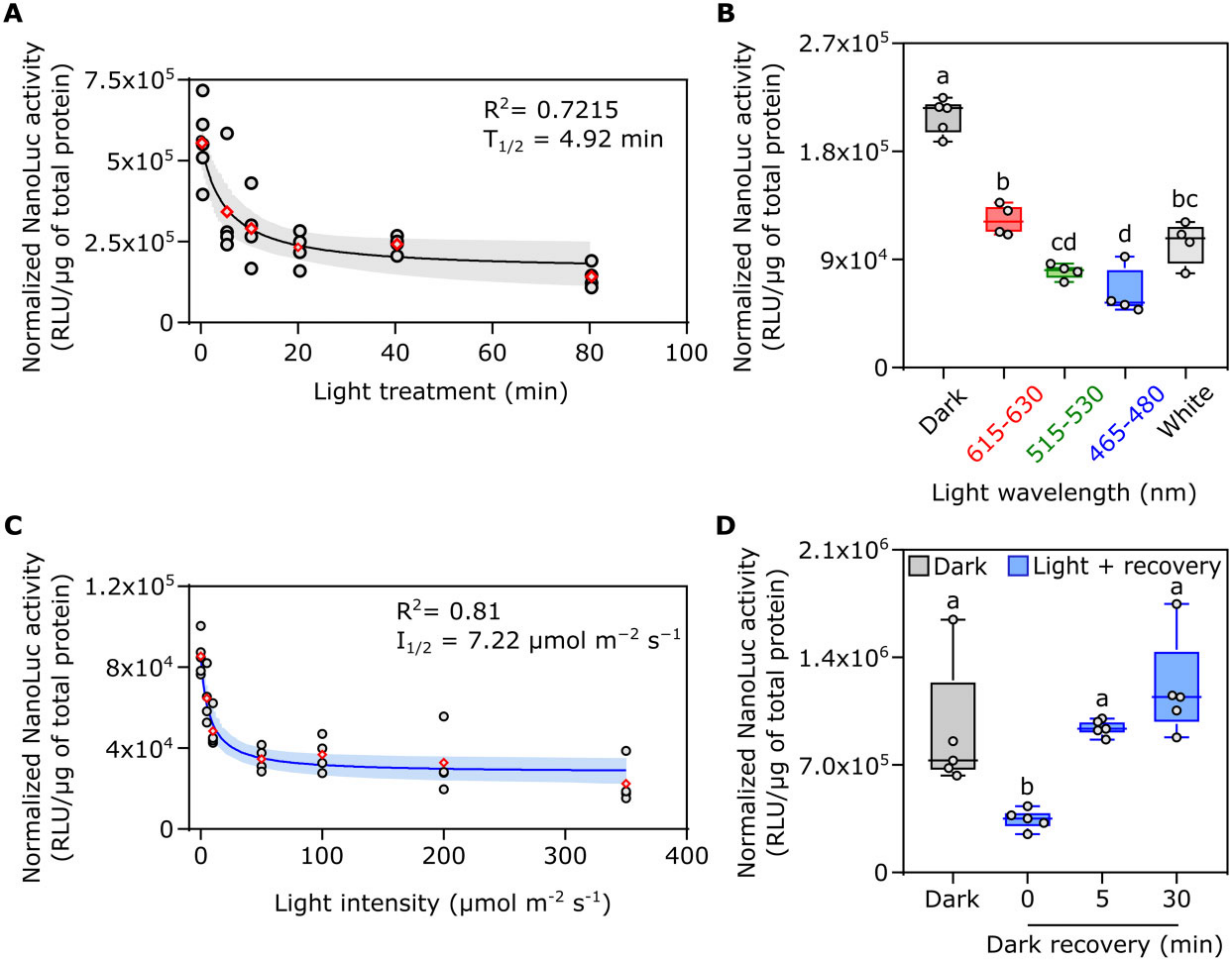
Characterization of the split-NLucOCP2 photoswitch in *A. thaliana* protoplasts. A, Impact of different lengths of light exposure (white light, 350 µmol m^−2^ s^−1^) on photoswitch output. Data points correspond to 0, 5, 10, 20, 40, and 80 min. B, Effect of different wavelengths on the output of the photoswitch, as compared to darkness. Red, green, blue, or white light was supplied for 20 min at 350 µmol m^−2^ s^−1^). C, Output modulation under increasing light intensity. Protoplasts were exposed to 5, 10, 50, 100, 200, or 350 µmol m^−2^ s^−1^ blue light for 20 min after 16-h dark incubation (initial data point). D, Test of the reversibility of the photoswitch. After light treatment (20 min, 50 μmol m^−2^ s^−1^, blue light), the samples were incubated for 5 or 30 min in the dark before sampling. In the box plots, dots represent biological replicates, the black line marks the median, and the box indicates the IQR. Whiskers extend to data points that are below 1.5× IQR away from the box extremities. Different letters indicate statistical differences (*P* ≤ 0.05) calculated from one-way ANOVA followed by Tukey’s post-hoc test (*n* ≥ 4). In dot plots, data fitting through nonlinear regression analysis is shown with blue continuous curves, generated using the GraphPad Prism built-in equation for [Inhibitor] versus response model. Shaded bands indicate the predicted 95% confidence interval. Black dots represent single data points and red rhombuses represent mean values. The *R*^2^, *T*_1/2_ of the curves are included in the graphs.

Once we found out the basal requirements of our photoswitch, we performed a full characterization of its dynamics, to describe how its activity could be modulated by the light. First, to understand the kinetic of the light-induced structural changes in the photoswitch, we treated the transformed *crtW* protoplasts for different periods with white light (350 μmol m^−2^ s^−1^), from 5 to 80 min, after dark incubation. Photoconversion of a significantly (one-way analysis of variance (ANOVA) followed by Tukey’s post-hoc test, *P* = 0.034) high amount of the complex occurred as early as 5 min after light exposure (*T*_1/2_ = 4.92 min) and increased further with time (Figure 4A). We selected 20 min light treatment for further analysis, since this data point was associated with a low variability among the biological replicates.

Next, we investigated whether the light quality is relevant for the regulation of our photoswitch activity, and which wavelength range is the most effective in triggering the switch. Treatments with a tuneable led lamp emitting red (615–630 nm), green (515–530 nm), blue (465–480 nm), or white light were compared with dark-treated samples (Figure 4B). We observed that the output was significantly different between the dark treatment and the whole set of different wavelengths: blue and green light exerted the strongest effect, while red light only halved the luminescent output. White light was not as effective as blue light to switch off the system, suggesting that saturated blue light is required to achieve the maximum photoswitch performance. However, we were puzzled by the repressive effect of red light on our switch, since carotenoids are unable to absorb that specific spectral range. A possible explanation for this observation implies the effect of red light on NLuc activity or stability. Thus, we generated a synthetic construct comprising the NNLuc and CNLuc fused together by the same linker used in the design of the OCP2-NLuc modules. This construct was cloned into a vector to be expressed in plant protoplasts under control of the 35S CaMV promoter and targeted to the chloroplast stroma. Two separate protoplast experiments were performed to test the effect of different light spectra on our synthetic NLuc: dark versus blue and green light (Supplemental Figure S4A) and dark versus red light (Supplemental Figure S4B) treatment. Neither blue nor green light affected Nanoluc activity, while we found a strong effect of red light, indicating that the reduction of the signal we saw in our switch under red light (Figure 4B) was not due to OCP2 photoactivation but to a general effect of red light on the synthetic NanoLuc. Based on these results, we avoided the use of white or red light for future experiments.

Having established the optimal treatment duration (20 min) and light spectrum (blue) to elicit photoswitch output, we moved on to analyze the effect of light intensity on our system, testing the range from 5 to 350 μmol m^−2^ s^−1^ (Figure 4C). We could measure a significant (one-way ANOVA followed by Tukey’s post-hoc test, *P* = 0.0006) signal decrease already at 10 μmol m^−2^ s^−1^. The response was saturated at 50 μmol m^−2^ s^−1^. To exclude a possible detrimental effect of the light treatments on protoplasts vitality, in particular after long (1 h and above) and high-intensity (350 µmol μm^−2^ s^−1^) treatments with blue light, the most energetic and potentially dangerous among those used, we carried out a time-course analysis, treating with blue light at 350 μmol m^−2^ s^−1^, where, in addition to *NLuc* expression and protein quantification, we also checked protoplasts vitality every 20 min, up to 80 min, through fluorescein diacetate staining. We found no significant difference in the percentage of viable protoplasts (Supplemental Figure S5A) between dark- and light-treated samples at each time point, while the photoswitch activity decreased over time under light (Supplemental Figure S5B), indicating that the lower luminescence in the light-treated protoplasts compared to the dark samples was caused by reduced enzyme activity rather than by decreased protoplast vitality.

Finally, we investigated the possibility that our synthetic photoswitch could revert from the dissociated to the dimeric form when light exposure ceases, as happens for heterodimeric OCP2 complexes in vitro (Lechno-Yossef et al., 2017). We shifted protoplast samples, previously exposed to 20-min saturating blue light (50 μmol m^−2^ s^−1^), to darkness for different amounts of time. We found out that the split-NLucOCP2 had the capacity to revert quickly to the dark-adapted conformation after the treatment, since we measured full recovery of the signal as early as 5 min into dark incubation (*T*_1/2_ = 2.79 min, Figure 4D).

### Stable transformation of the split-NLuc-OCP2 photoswitch in Arabidopsis plants

**Figure 5:**
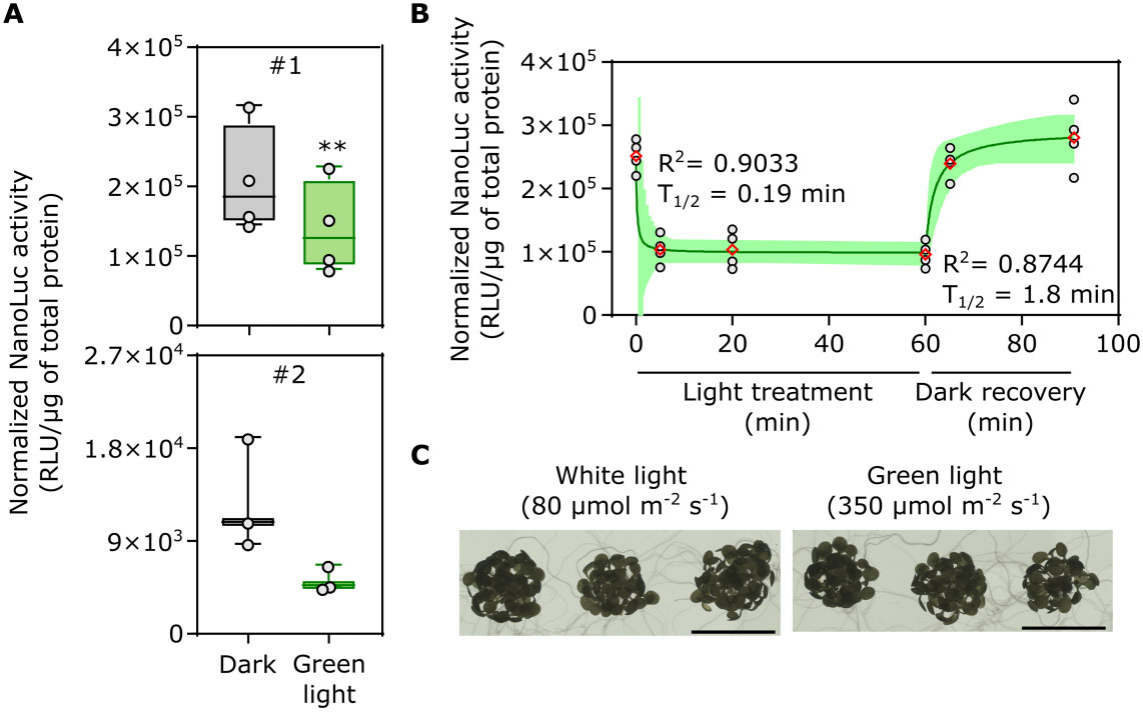
Characterization of the split NLuc-OCP2 photoswitch in *A. thaliana* stably transformed plants. A, Luminescence of two independent lines (#1, #2) carrying both the PLS-CNLuc-CTD and the PLS-NTD-NNLuc modules, treated with dark or green light (20 min, 350 µmol m^−2^ s^−1^). B, Effect of different duration of light exposure (green light, 350 µmol m^−2^ s^−1^) and dark recovery on line#1 luminescence. Data points correspond to 0, 5, 20, and 60 min of light exposure, followed by 5 and 30 min of dark recovery. In the box plots, dots represent biological replicates, the black line marks the median, and the box indicates the IQR. Whiskers extend to data points that are below 1.5× IQR away from the box extremities. A Student *t* test was used to assess significant differences. Asterisks indicate statistical differences (*P* ≤ 0.01, *n* ≥ 3). In the dot plot, data fitting through nonlinear regression analysis is shown by a green continuous curve. Shaded bands indicate the predicted 95% confidence interval. Black dots represent single data points and red diamonds represent mean values. The *R*^2^ and *T*_1/2_ of the curves are included in the graphs. C, Comparison of seedlings treated with high-intensity green light for 1 h or with low-intensity white light. No difference was visible.

The performance of split-NLucOCP2 in transiently transformed protoplasts prompted us to validate these results with stable transformant plants. We thus generated binary plasmids to express *PLS-NTD-NNLuc* (under the control of the 35S CaMV promoter) and *PLS-CNLuc-CTD* (UBQ10 promoter) and transformed *crtW#11* plants with these. We selected two independent lines carrying both the *PLS-CNLuc-CTD* and the *PLS-NTD-NNLuc* modules which showed luminescence in the dark (Figure 5A), indicating that the two modules can associate also when stably expressed in planta, confirming our previous results obtained through transient transformations. To investigate the kinetics of light activation and dark reversion of the split-NLucOCP2 in stable-transformed plants, we performed a time-course analysis with green light at 350 µmol m^−2^ s^−1^. Eight-day-old seedlings showed a significant (one-way ANOVA followed by Tukey’s *post* *hoc* test, *P* = 0.0001) reduction in luciferase activity after 5-min light exposure. Longer green light exposure did not decrease this activity further, indicating stimulus saturation. Moreover, 5-min dark incubation were sufficient to fully recover the signal measured before the light treatment (Figure 5B). We also assessed whether high-intensity green light exerted any detrimental effect on these young seedlings. We could not spot any difference with untreated seedlings (Figure 5C). In the light of these observations, we concluded that our OCP-based photoswitch performed similarly when expressed transiently or stably in planta.

### Application of the OCP2-based photoswitch to control gene expression in chloroplasts

**Figure 6:**
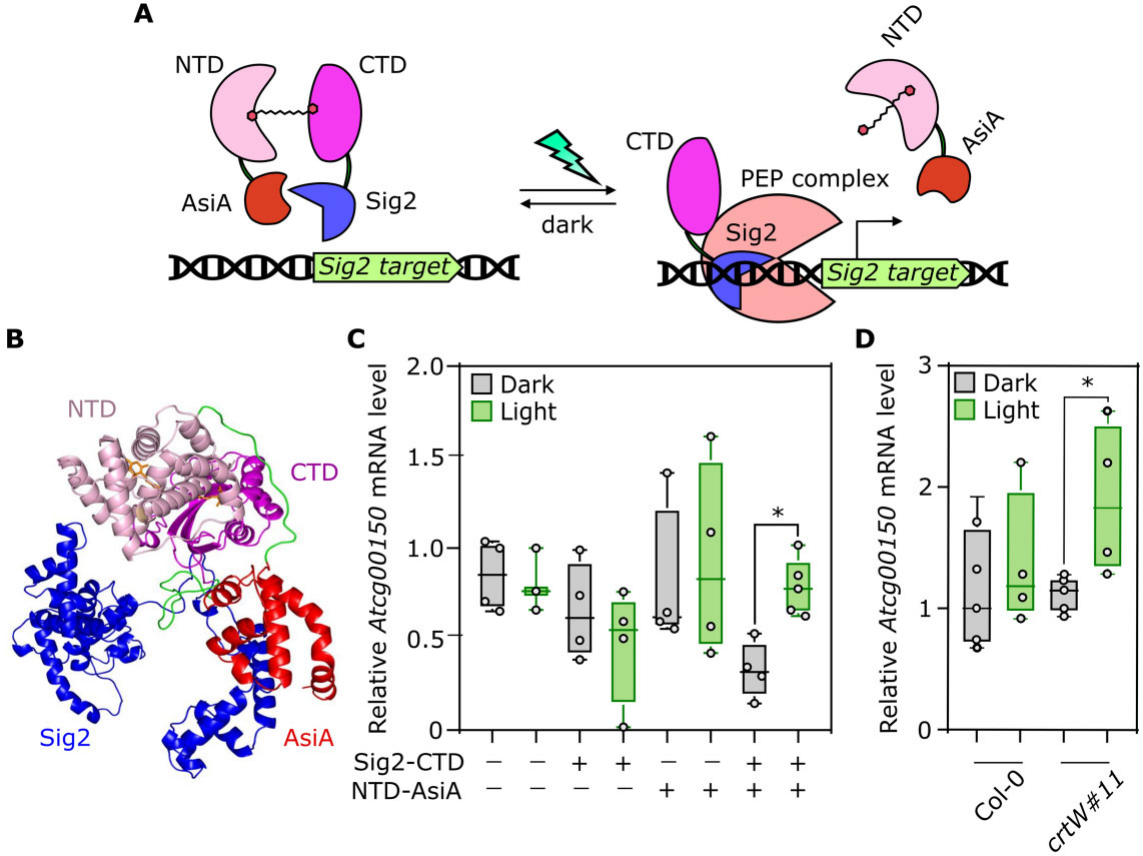
Design and validation of a strategy to control chloroplast gene expression in response to blue–green light. A, Scheme of the desired genetic circuit, based on association/dissociation of the DNA–RNA polymerase complex. On the left, the association between the two OCP2 modules in the dark results in the interaction between Sig2 and AsiA, preventing PEP–Sig2–DNA association by steric hindrance. On the right, after blue–green light treatment, AsiA dissociation allows Sig2 to recruit the PEP complex and consequently to induce gene transcription. B, In silico prediction of Sig2–CTD and NTD–AsiA interaction in the presence of the keto-carotenoid. C, RNA level of the Sig2 target *Atcg00150* in response to darkness or green light (350 µmol m^−2^ s^−1^) for 4 h in *crtW* leaves infiltrated with different combinations of agrobacteria bearing the constructs Sig2–CTD and NTD–AsiA. D, Comparison of *Atcg00150* transcript between Col-0 and *crtW* plants agroinfiltrated with Sig2–CTD and NTD–AsiA after a 4-h dark or green light (350 µmol m^−2^ s^−1^) treatment. A Student *t* test was used to assess significant differences. Asterisks indicate statistical differences (*P *≤ 0.01, *n* = 4). In the box plots, dots represent biological replicates, the black line marks the median, and the box indicates the IQR. Whiskers extend to data points below 1.5× IQR away from the box extremities.

Relying on the characterization carried out with NLuc fusions, we moved on to exploit our OCP2-based photoswitch for biotechnological applications. The dissociative nature of our construct directed us to envisage a double negative regulation to activate a process in response to the light stimulus. We set out to engineer a strategy to control chloroplast gene expression in response to blue–green light. Gene transcription in these organelles is operated by the nuclear-encoded RNA polymerase and plastid-encoded polymerase (PEP; Chi et al., 2015). The latter is a protein complex which includes nuclear-encoded factors that share high sequence similarity with bacteria sigma 70 (σ^70^) factors, responsible for polymerase docking onto promoter sequences (Tripathi et al., 2014). In prokaryotes, polypeptides that inhibit sigma association with the RNA polymerase holoenzyme or DNA are defined as anti-sigma factors (Hughes and Mathee, 1998). We reasoned that, given the high similarity between sigma factors in bacteria and plants, anti-sigma factors might retain repressive activity against the latter. Thus, following our established protocol for structure-guided design of protein fusions, we generated a possible 3D model where the OCP2 CTD, fused to the C-terminus of an Arabidopsis Sigma2 (Sig2) factor (Börner et al., 2015), and the NTD, fused to the N-terminus of the T4 phage anti-sigma factor AsiA (Orsini et al., 1993), prevent PEP–Sig2–DNA association by steric encumbrance (Figure 6, A and B). We hypothesized a weak interaction between Sig2 and AsiA, due to the conservation, in Sig2, of some of the residues of *Escherichia coli* σ^70^-type ropD required the interaction with AsiA (Supplemental Figure S6). This interaction might be enhanced when the two proteins are forced in proximity by the interaction of additional domains, as in our case OCP2 NTD and CTD would do. We set out to test our hypothesis by testing the effect of green light on the Sig2 target *ATPI* (*Atcg00150*) (Ghulam et al., 2012) in cells expressing both fusion proteins. Due to the limited amount of RNA extractable from protoplast samples, we resorted to the transient transformation of mesophyll cells by agroinfiltration of *crtW* plants. Expression of either construct did not significantly alter the expression of the Sig2 target, while simultaneous transformation repressed its expression in the dark. Exposure to green light (350 µmol m^−2^ sec^−1^) for 4 h restored ATPI expression to wild-type levels (Figure 6C). To verify that the effect on Sig2 activity was OCP2-dependent, we repeated the experiment by transforming wild-type plants, where no keto-carotenoid is produced. We confirmed ATPI induction in the light in *crtW* #11 plants, but not in the wild type (Figure 6D), indicating that the Sig2-CTD and NTD-Asia interact only in the presence of the keto-carotenoid and that green light is sufficient to break this interaction apart, thus releasing the chimeric Sig2 to be able to participate to the PEP complex.

## Discussion

In this work, we successfully exploited the properties of a cyanobacterial OCP2 to generate a synthetic photoswitch meant to be active in plant chloroplasts. We drew a first characterization of a prototype version of our construct in living protoplasts and subsequently confirmed its properties in stably transformed plants. As according to our design (Figure 1D), reconstitution of a functional luciferase, based on the association of NTD and CTD of OCP2 fused to NLuc fragments, was prevented by blue–green light (Figure 3A). Moreover, the photoswitch was able to revert spontaneously to the heterodimeric state in the dark (Figure 4D). The extent of luciferase inhibition was directly proportional to the treatment duration and light intensity (*T*_1/2_ 4.92 min and *I*_1/2_ 7.22 μmol m^−2^ s^−1^, Figure 4, A and C), with slower kinetics than those measured for *Fremyella* split-OCP2 heterodimer formation (*T*_1/2_ of 1.7 min at 23°C, Lechno-Yossef et al., 2017). Altogether, this evidence led us to conclude that split OCP2 strategies are viable to design fast-responding and quickly reversible photoswitches, characterized by a continuous response to light and a high dynamic range. As expected the blue and green ranges of the light spectrum were the most effective in switching off the response (Figure 4B). The effect of red light to inactivate our photoswitch, however, was unexpected, since OCP2 has an absorption peak in the blue–green range at 474 and 502 nm and cannot be photoactivated by red light (Bao et al., 2017). Indeed, tests on a synthetic NanoLuc construct comprising the NNLuc and the CNLuc fragments fused by the same linker used in the design of the split-NLuc/OCP2 modules uncovered specific signal repression by red light (Supplemental Figure S4B), while blue and green light did not affect the output (Supplemental Figure S4A). Although outside of the scope of our specific study, this observation deserves further study and characterization in the future, to understand whether red light impacts protein stability or activity, the dynamics of such inhibition and the molecular mechanism that enables it.

Two features proved to be essential for the synthetic photoswitch to control luciferase activity: first, chloroplasts needed to produce the required keto-carotenoid prosthetic group and second, the two photoswitch modules required plastidial localization. Thus, different from already established systems based on naturally available prosthetic groups, we had to implement a canthaxanthin biosynthetic pathway by expressing an exogenous β-carotene ketolase (crtW) enzyme (Figure 2). Indeed, when protoplasts expressed cytosol-localized modules (Figure 3A) or were unable to produce keto-carotenoids (Figure 3B), the switch was nonfunctional. Dimerization and photoactivation of the photoswitch could be related to either canthaxanthin or astaxanthin, which in both cases were recently demonstrated to be bound by OCP2, in the case of heterologous expression of this subunit in the green alga *Chlamydomonas reinhardtii* engineered for producing keto-carotenoids (Pivato et al., 2021). In the keto-carotenoid producing plants, we found that astaxanthin was reproducibly more abundant than canthaxanthin (Figure 2F; Supplemental Table S1). This is not entirely surprising, given previous demonstrations that canthaxanthin, generated by action of ketolase enzyme on β-carotene can be hydroxylated to astaxanthin by endogenous hydroxylases and zeaxanthin can be directly converted to astaxanthin by ketolases (Zhong et al., 2011; Bai et al., 2017). Comparison of the composition of the carotenoid pool in *ctrW* and wild-type plants showed that keto-carotenoids were synthesized at the expense of other β–β xanthophylls. As shown in Supplemental Table S1, neoxanthin, β-carotene and violaxanthin were less abundant in keto-carotenoids producing lines, even though only the latter shows a significant decrease. This was expected because β-carotene and zeaxanthin are substrates of the crtW enzyme, but also precursors of both neoxanthin and violaxanthin (Figure 2A). On the other hand, ε–β xanthophyll lutein, whose biosynthesis follows a different branch of the pathway (Ruiz-Sola and Rodríguez-Concepción, 2012; Bai et al., 2017), was unaffected (Figure 2A).

Light exposure could not abolish OCP2-nanoLuc signal entirely, even after several hours. The signal still present in light-treated *crtW* protoplasts (Figure 3A) can be explained by two nonmutually exclusive hypotheses: that the treatment could not separate the whole pool of interacting NTD–CTD modules, or that other carotenoids, such as β-carotene, may mediate interaction, although without the ability to photoconvert (Punginelli et al., 2009; Wilson et al., 2011). Recently, *F.* *thermalis* OCP2 was shown to bind neoxanthin, loroxanthin, and lutein when heterologously expressed in *C.* *reinhardtii* (Pivato et al., 2021). The latter scenario could also explain the amount of signal we measured in dark-treated wild-type protoplasts, which was not affected by the light treatment (Figure 3A). Moreover, the complete absence of signal observed in the cytosol-localized photoswitch suggests that none of the carotenoids able to conjugate with OCP2 were present outside the chloroplast in sufficient amounts to associate with the photoswitch (Figure 3B). To improve the dynamic response and reduce the basal activity of the switch under light conditions, random or structure-informed mutagenesis of the OCP2 modules could be applied to tune the affinity between the NTD and the CTD. For example, mutagenesis studies on the OCP1 protein led to the identification of amino acid substitutions that altered keto-carotenoid specificity, ability to photo-convert and stability of the red form (OCP-R; Leverenz et al., 2015; Wilson et al., 2011). However, some of the residues that determine OCPs activity are not conserved among OCP families (Bao et al., 2017), thus limiting consequence predictability for substitutions in OCP2.

The success of this initial study lays the foundations for different ways in which our system could be exploited in the future. Like NLuc, the OCP2 modules can be fused to a bipartite enzyme or structural protein whose function is favored under dark conditions and/or not required or even detrimental when cells are illuminated. In this work, we applied the split-OCP2 strategy to control reversible association of an anti-sigma factor from T4 phage AsiA with Sig2, one of the five sigma subunits of the PEP that transcribes plastid genes. Structure-informed design of the fusion between the OCP2 modules and the two regulatory proteins to be tested transiently in *crtW* plants. This construct successfully enabled inhibition of a Sig2 target gene (*ATPI*) in the dark and release of repression when plants were exposed to green light (Figure 6B). This result sets the basis to extend application of the split-OCP2 strategy to other sigma factors (Lysenko, 2007), to control the expression of other plastid genes. While presenting a limitation for use in cells that are not able to synthesize keto-carotenoids, since these cannot be provided exogenously due to their lipophilic nature, our system could be exploited not only for its photo-induced activity, but also as a carotenoids biosensor to detect their metabolism in wild-type and metabolically engineered cells: when carotenoids without keto-groups are present in the cellular environment, a small percentage of the modules is expected to dimerize in a stable manner; instead, if in case of keto-carotenoids accumulation, the dimerization rate will be higher and reversible under light treatment (Figure 3A). Alternatively, this photoswitch strategy might be applied in microalgae, where different species accumulate keto-carotenoids or can be successfully engineered to express them (Perozeni et al., 2020). An additional limitation of our OCP2-based photoswitch is its activation under conventional photoperiodic conditions that entail white or blue light, leading to on/off fluctuations during the day/night cycles. While this may represent a drawback for timely and tight control of the photoswitch output, this feature could be exploited to selectively control gene expression during either day or night, for example regulating metabolic processes that are beneficial only during the day while detrimental during the night, or vice versa.

In summary, here, we demonstrated that plant organelles can be equipped with an orthogonal synthetic photoswitch, based on a cyanobacterial light-responsive protein and its cognate keto-carotenoids. The behavior of this synthetic construct led us to conclude that, the structural modularity of OCP, together with its light-driven dissociation (Leverenz et al., 2014; Lechno-Yossef et al., 2017), makes it a suitable tool for optogenetic applications (Dominguez-Martin and Kerfeld, 2019). Selecting biological modules from unrelated species grants the highest probability that cross-interactions between components and endogenous pathways are kept to a minimum, and we consider particularly interesting the ability of OCPs to be triggered by the green range of the light spectrum, considering that it should not interfere and stimulate the most common photoreceptors already known inside the plant systems, ensuring the orthogonality of the system. In conclusion, this system holds great potential for future applications in prokaryotes and eukaryotic cells to control gene expression and enzyme activity in response to blue–green light.

## Materials and methods

### Design of synthetic DNA sequences and plasmid assembly

Sequences were designed, codon-optimized for Arabidopsis (*A.* *thaliana*) expression, and synthesized as DNA strings using the GeneArt service (Thermo-Fisher Scientific, Waltham, MA, USA). The sequences of the synthetic constructs devised for this work are provided in Supplemental Information S1. Directional cloning of DNA fragments was performed using the pENTR Directional TOPO Cloning Kits (Thermo-Fisher Scientific). For the OCP2 modules we took the *F.* *thermalis* OCP2 sequence as reference (accession WP_009459388). The nonchloroplast localized fragments were amplified with the Phusion High-fidelity DNA polymerase (Thermo-Fisher Scientific) using the following primer couples: NTD-NNLuc-Fw (CACCATGTCCTTCACCATCGAG) with NTD-NNLuc-Rv (TCAGCTGTTGATGGTCACTCTG) and CNLuc-CTD-Fw (CACCATGGTGACCGGGTACA) with CNLuc-CTD_Rv (TCACTGGATGAATCCCATGTTG). The resulting entry vectors were then recombined into destination vectors via Gateway LR Clonase II Enzyme mix (Thermo-Fisher Scientific), to produce the desired expression constructs. A 35S:*PLS-crtW* construct for constitutive transgene expression in plants was obtained by recombination of the proper entry vector, containing the *crtW* coding sequence from *A.* *aurantiacum* (*Paracoccus sp.* N81106), equipped with the PLS of *P.* *sativum* RBCS-3, with the binary vector pK7WG2 (Karimi et al., 2002). The individual modules PLS-NTD-NNLuc, PLS-CNLuc-CTD, NTD-NNLuc, and CNLuc-CTD were, instead, recombined with the plasmid p2GW7 (Karimi et al., 2002), suitable for transient expression in Arabidopsis protoplasts. The 35S:Sig2-CTD expression plasmid was generated as follows. Sig2 CDs was cloned using the oligonucleotides Bp_Sig2_Fw (AAAAAAGCAGGCTCCATGTCTTCTTGTCTTCTTCC) and Sig2_Link_Rv (CCACCTCCGCTAGATCCGGATGATTGTGCAACCAAGTATTG) with the plasmid TOPO-U15-A03 (ABRC) as a template, and linker-CTD fragment was produced using the oligonucleotides linker_CTD_Fw (TCCGGATCTAGCGGAGGTGG) and BP_CTD_Rv (AGAAAGCTGGGTGTCACTGGATGAATCCC) using PLS-CNLuc-CTD as template. These two fragments were fused by overlapping PCR using Bp_Sig2_Fw and BP_CTD_Rv and subcloned in pDNR201 (Invitrogen Waltham, MA, USA), followed by recombination in the pB7WG2 destination plasmid (Karimi et al., 2002) by Gateway technology using the LR Clonase II Enzyme mix (Thermo Fisher Scientific). For the pUBQ:PLS-NTD-ASIA expression plasmid, The UBQ10 promoter was amplified by PCR from pUB-DEST using primers pHindIII-UBQ10fw (gggaagctttacccgacgagtcagta) and pUBQ10-SpeIrv (cccactagtagtgttaatcagaa) and ligated into SpeI/HindIII-digested pH7WG2. The orientation and sequence of the UBQ10 promoter in the resulting pH7WGUbq10 plasmid was verified by Sanger sequencing. A DNA string containing the entire CDS encoding PLS-NTD-AsiA (Supplemental Information S1), surrounded by attB1 and attB2 (Gene art gene synthesis-Invitrogen), sites were first subcloned in the pDNR201 and later recombined in the pH7WGUbq10.

### Protein structure modeling

The 3D structures were generated as follows. Amino acid sequences were first aligned to template sequences using MultAlin (Corpet, 1988) or MEGA (Tamura et al., 2021) software and the resulting alignment used as input file for modeling using Modeller (Webb and Sali, 2016). For both CNluc‐CTD and NTD‐NNluc sequences of OCP of *A.* *maxima* (PDB: 5UI2) (Kerfeld et al., 2003) and the synthetic luciferase NanoLuc (PDB: 5IBO) (Lovell et al., unpublished data) were used as templates. For Sig2-CTD, the sequences of OCP of *A. maxima* (PDB: 5UI2; Kerfeld et al., 2003), RNA polymerase sigma factor SigA of *Mycobacterium tuberculosis* (PDB: 5UI2, 6BZO, and 7KIF; Lin et al., 2017; Boyaci et al., 2018; Lilic et al., 2021), and RNA polymerase sigma factor SigA of *Bacillus subtilis* (PDB: 7CKQ; Fang et al., 2020) were used as template. For NTD-ASIA, the sequences of OCP of *A. maxima* (PDB: 5UI2) (Kerfeld et al., 2003), Escherichia virus T4 Anti-Sigma Factor AsiA Homodimer (PDB: 1JR5, 6K4Y; Urbauer et al., 2002) were used as templates. Figure rendering was performed with the PyMol software (DeLano, 2020).

### Plant materials and growth conditions

Experiments were carried out using *A.* *thaliana* accession Col-0 as the wild-type background. The *crtW* over-expressors were obtained exploiting *Agrobacterium tumefaciens*-mediated transformation of the 35S:PLS-crtW construct in Col-0, following the floral dip protocol (Clough and Bent, 1998). Transgenic seedlings were selected for resistance on kanamycin and subsequently verified by PCR for the presence of the transgene with the following primers: attB1 (GGGACAAGTTTGTACAAAAAAGCAGGCT) and PLS_crtW_Rv (TCAGGCGGTATCACCCTTAGT). Soil-grown plants were cultivated at 23°C day/18°C night under neutral day photoperiod (12-h light:12-h darkness) in single 6 cm pots using a 3:1 soil: perlite mixture (with HAWITA tray substrate), after seed vernalization at 4°C in the dark and germinated. The quantum irradiance was 80- to 100-μmol photons m^−2^ s^−1^. For the in vitro selection of transformed plants, seeds were surface sterilized using 70% (v/v) ethanol and 10% (v/v) commercial bleach solution and then rinsed 5–7 times with sterile distilled water. Seeds were then sown on solid sterile 1/2 strength Murashige–Skoog (MS) medium [0.215% (w/v) MS salts (Sigma-Aldrich), 0.8% agar (w/v), 0.5% (w/v) sucrose, pH 5.7]. Genomic DNA was extracted following the protocol described by Edwards et al. (1991).

### RNA extraction and gene expression analysis by RT-qPCR

For RT-qPCR analysis, total RNA was extracted from 4-week-old plants as described by Kosmacz et al. (2015). cDNA was synthesized from 1 μg of total RNA using the Maxima Reverse Transcriptase kit (Life Technologies, Carlsbad, CA, USA). Real-time PCR amplification was performed on 12.5-ng cDNA with the ABI Prism 7300 sequence detection system (Applied Biosystems, Waltham, MA, USA), using the PowerUp SYBR Green Master Mix (Applied Biosystems). Ubiquitin10 (*At4g053290*) was exploited as the housekeeping gene (Czechowski et al., 2005). A pair of specific primers was designed on the *crtW* gene sequence: sgCRTWfw (GGCACAACGCTCGTTCCTCT) and sgCRTWrv (AAACGCCACCAAGGCACAGT). The primers used to monitor the expression of *Atcg00150* are SgAtcg00150Fw (TCTAGCGATTCGCAATCCACAAAC) and SgAtcg0050Rv (GGGAACCCATGGACCATATTCTTC).

### Plasmid DNA purification

Highly concentrated plasmid DNA required for protoplast transformation was extracted from 100-mL bacterial culture (Luria-Bertani [LB] medium) supplemented with the appropriate antibiotic. Bacterial pellets were extracted with an alkaline lysis protocol, according to Sambrook and Russell, 2001. Briefly, pellets were sequentially resuspended in 2 mL of a buffer containing 50-mM Glucose, 25-mM Tris–HCl, and 10-mM EDTA (pH 8), lysed in 4 mL of a buffer containing 0.2-M NaOH and 1% (w/v) sodium dodecyl sulfate (SDS) and neutralized in 3 mL of a buffer composed of 3-M potassium acetate in 11.5% (v/v) glacial acetic acid. Next, nucleic acids were precipitated with an equal volume of isopropanol and then treated with RNase A (Sigma-Aldrich) for 1 h at 37°C. Following polyethylene glycol (PEG) precipitation (13% [w/v] PEG 8000 dissolved in 1.6-M NaCl), phenol:chloroform extraction was performed and nucleic acids were precipitated in absolute ethanol, with the aid of ammonium acetate 0.6 M. Final elution was in nuclease-free water.

### Protoplast isolation and transformation

Arabidopsis mesophyll protoplasts were isolated and transformed according to Wu et al. (2009) with some modifications. Leaves of 4-week-old plants were deprived of the lower epidermis using paper tape, immersed in 10 mL of enzymatic solution (0.4-M mannitol, 20-mM KCl, 20-mM MES, pH 5.7, 10-mM CaCl_2_, 1% [w/v] cellulase, and 0.4% [w/v] macerozyme), and left at 23° in the dark for 2.5 h. The released protoplasts were washed with W5 solution (154-mM NaCl, 125-mM CaCl_2_, 5-mM KCl, and 2-mM MES) and filtered with a mesh. They were then pelleted at 100 g for 1 min, resuspended with W5 and kept for 20-min pelleting in the dark before resuspension in MMG solution (0.4-M mannitol, 15-mM MgCl_2_, and 5-mM MES) to a final concentration of 5 × 10^5^ cells mL^−1^. About 100 µL of protoplasts were mixed with 3 µg of each effector plasmid and 110 µL of freshly prepared solution of 40% (w/v) PEG4000, 100-mM CaCl_2_, and 200- mM mannitol. After 20 min of dark incubation, 440 µL of W5 were added to the mix, the protoplasts were pelleted for 1.5 min at 200 g and resuspended with 500 µL or 1 mL of WI (500-mM mannitol, 4-mM MES, and 20-mM KCl). Protoplasts were incubated in 6- or 24-multiwell plates at 23°C in the dark for 16 h. Unless differently stated, protoplasts isolated from the *crtW#11* line were used for the experiments.

### Agrobacterium-mediated infiltration of Arabidopsis leaves

Agrobacterium infiltration of Arabidopsis leaves was performed using the protocol described by Panicucci et al. (2021) with some modifications. *Agrobacterium tumefaciens* cultures (strain GV3101) were grown overnight in LB media supplemented with proper antibiotics and 20-μM acetosyringone. The cultures were then pelleted, and the bacteria were resuspended in MMA solution (MS 5 g L^−1^, MES 1.95 g L^−1^, sucrose 20 g L^−1^, pH 5.6) supplemented with 200-μM acetosyringone until reaching a final optical density (OD_600_) of 0.4. When two Agrobacterium were used in combination, the two cultures were mixed in a 50:50 ratio. Agrobacterium cultures were then grown at room temperature for 3–4 h and finally used to infiltrate the abaxial side of 3-week-old Arabidopsis leaves. Agroinfiltrated plants were kept in the dark overnight (O.N.) and later moved to normal photoperiod for another 24 h. Plants were finally moved to dark or green light for 4 h and later collected in liquid nitrogen for RNA extraction.

### Light treatment of transfected protoplasts

Multiwell plates containing the transfected protoplasts were placed under LED Floodlight Outdoor 40 W lamp (Jayool), with the possibility to change both light wavelength and intensity. Red (615–630 nm), green (515–530 nm), blue (465–480 nm), and white light were used in our experiments, testing an intensity range from 5 to 350 μmol m^−2^ s^−1^. For the analysis of the reversion kinetics, the light-treated samples were transferred in darkness for 5 or 30 min before sampling.

### Luciferase activity quantification

Protoplasts were flash frozen in liquid nitrogen and lysed by adding 30 μL of Passive Lysis Buffer (Promega, Madison, WI, USA). NanoLuc activity was then measured using the Nano-Glo Luciferase Assay (Promega) following the manufacturer’s instructions. Luminescence was detected with a tube Luminometer (Lumat LB 9507 Berthold) using an integration time of 10 s. NanoLuc measurements were normalized according to the total protein concentration of each sample, determined by means of the Bio-Rad colorimetric assay, based on the Bradford dye-binding method (Bradford, 1976).

### Assessment of protoplast viability

About 600 µL of transfected protoplasts were aliquoted in 24-multiwell plates. At each time point 99 µL of the treated protoplasts were mixed with 1 µL of fluorescein diacetate (1 µg mL^−1^; Sigma-Aldrich), incubated 10 min in the dark, and imaged with a Leica THUNDER Imager Model Organism stereo microscope. Viability was calculated as the percentage of fluorescein diacetate-stained intact protoplasts. The remaining 500 µL of protoplasts were then used for luciferase activity quantification.

### Extraction and quantification of carotenoid content

Pigments were extracted from leaves of 6-week-old plants with 80% v/v acetone buffered with Na_2_CO_3_ and quantified by reverse-phase HPLC as described in Perozeni et al. (2020). A Jasco LC-4000 extrema HPLC system equipped with a C18 column (Synergi 4u Hydro-RP 80A, Phenomenex, USA) was used. Fifteen-minute gradient of ethyl acetate (0%–100%) in acetonitrile–water–triethylamine (9:1:0.01, v/v/v) at a flow rate of 1.5 mL/min was used (Lagarde et al., 2000) and pigment detection was conducted with a Jasco 350- to 750-nm diode array detector. Keto-carotenoids peaks were identified by comparing retention times and spectra to commercially available standards (CaroteNature GmbHas) as reported in Perozeni et al. (2020).

### Phenotypic analysis

To quantify the phenotypic parameters of interest in *crtW* plants, pots were imaged at 17, 21, and 24 d after germination with a LabScanalyzer (LemnaTec, GmbH, Aachen, Germany), and images were taken and analyzed as described by Ventura et al. (2020).

### Statistical analyses

Significant variations between genotypes or treatments were statistically evaluated using Student’s t test and ordinary one-way ANOVA with multiple comparisons. Curves were fit to the data with linear/nonlinear regression using the GraphPad Prism built-in equation for [Inhibitor] versus Response model. All the analyses were performed with GraphPad Prism version 9 for Windows version 10. For the plot shown in Figure 5B, the curves for light treatment and dark recovery were generated separately.

### Accession numbers

Sequence data from this article can be found in Supplemental Dataset 1.

## Supplemental data 

The following materials are available in the online version of this article.

**Supplemental Figure S1.** Alignment of the *F.* *thermalis* OCP2 (FtOCP2) and *A.* *maxima* OCP (AmOCP).

**Supplemental Figure S2.** Phenotypic comparison between Col-0 and keto-carotenoid producing plants.

**Supplemental Figure S3.** Comparison of split-NlucOCP2 activity in two different *crtW* lines.

**Supplemental Figure S4.** Effect of different light spectra on the activity of a synthetic NanoLuc.

**Supplemental Figure S5.** Comparison between protoplast vitality and Nanoluc activity in light treated samples.

**Supplemental Figure S6.** Multialignment of the 4.3 region (shaded in gray) of *A. thaliana* Sig2 and E. coli RpoD.

**Supplemental Table S1.** Comparison of the carotenoid content in *crtW* and wild-type Arabidopsis plants.

**Supplemental Dataset 1.** Nucleotide sequences of the constructs used in this work.

## Supplementary Material

kiac122_Supplementary_Data

## Notes

B.G., F.L., and L.P. conceived the project design and testing strategy. M.B., B.G., and F.L. supervised the experiments. L.P. and S.I. generated and analyzed transgenic plants, cloned the constructs, and tested them, S.C. conducted the carotenoid analyses, and S.I. modeled protein structures. L.P. and S.I. analyzed the data. L.P., B. G., and F.L. wrote the article with contributions of all the authors. F.L. agrees to serve as the author responsible for contact and ensures communication.

The author responsible for distribution of materials integral to the findings presented in this article in accordance with the policy described in the Instructions for Authors (https://academic.oup.com/plphys/pages/general-instructions) is Francesco Licausi (francesco.licausi@plants.ox.ac.uk).
