# Supplementary material for: A synthetic switch based on orange carotenoid protein to control blue–green light responses in chloroplasts
Source: Plant Physiol. 2022 Mar 15;189(2):1153–68. doi: 10.1093/plphys/kiac122 (PMC9157063; doi:10.1093/plphys/kiac122)
Supplement: kiac122_Supplementary_Data [file kiac122_supplementary_data.pdf]

```
FtOCP2      MSFTIESARSIFPETEVANAIPAIVESFNQLSAEDQLALLWFAYTEMGVTITPAAMGAAN    60
AmOCP       MPFTIDTARSIFPETLAADVVPATIARFKQLSAEDQLALIWFAYLEMGKTITIAAPGAAN    60
            * ***::******** .*:.:** :  *:**********:**** *** *** ** ****

FtOCP2      MVFAEKTLTQIKQMSAREQTQVMCDLINHADTPICRTYSSFGTNVKLGFWYQLGEWMKQG    120
AmOCP       MQFAENTLQEIRQMTPLQQTQAMCDLANRTDTPICRTYASWSPNIKLGFWYELGRFMDQG    120
            * ***:** :*:**:  :***.**** *::********:*:. *:******:**.:*.**

FtOCP2      IVAPIPEGYKLSAQASDVLQTIRQLEGGQQLTVLQNIVVNMGYAPTV--SAQKVKEPVVP    178
AmOCP       LVAPIPEGYKLSANANAILVTIQGIDPGQQITVLRNCVVDMGFDTSKLGSYQRVAEPVVP    180
            :************:*. :* **: :: ***:***:* **:**:  :   * *:* *****

FtOCP2      PKDIAPRAKVSIEGIDNLTVLSYMENMNAFDFQGAVALFAENGALQPPFQDPIVGQENIL    238
AmOCP       PQEMSQRTKVQIEGVTNSTVLQYMDNLNANDFDNLISLFAEDGALQPPFQKPIVGKENTL    240
            *:::: *:**.***: * ***.**:*:** **:. ::****:********.****:** *

FtOCP2      AYMREECYGLKLIPERGISEPAEGGFTQVKVTGKVQTPWFGDSVGINLAWRFLLNPQGKI    298
AmOCP       RFFREECQNLKLIPERGVSEPTEDGYTQIKVTGKVQTPWFGGNVGMNIAWRFLLNPENKV    300
             ::**** .********:***:*.*:**:************..**:*:********:.*:

FtOCP2      FFVAIDVLATPQELLNMGFIQ 319
AmOCP       FFVAIDLLASPKELLNL---- 317
            ******:**:*:****:
```

Supplemental Figure S1. Alignment of the *Fischerella thermalis* OCP2 (FtOCP2) and *Arthrospira maxima* OCP (AmOCP). Asterisks residue indicate identity between the two sequences; ‘:’ and ‘.’ indicate similar physical-chemical properties of residues.

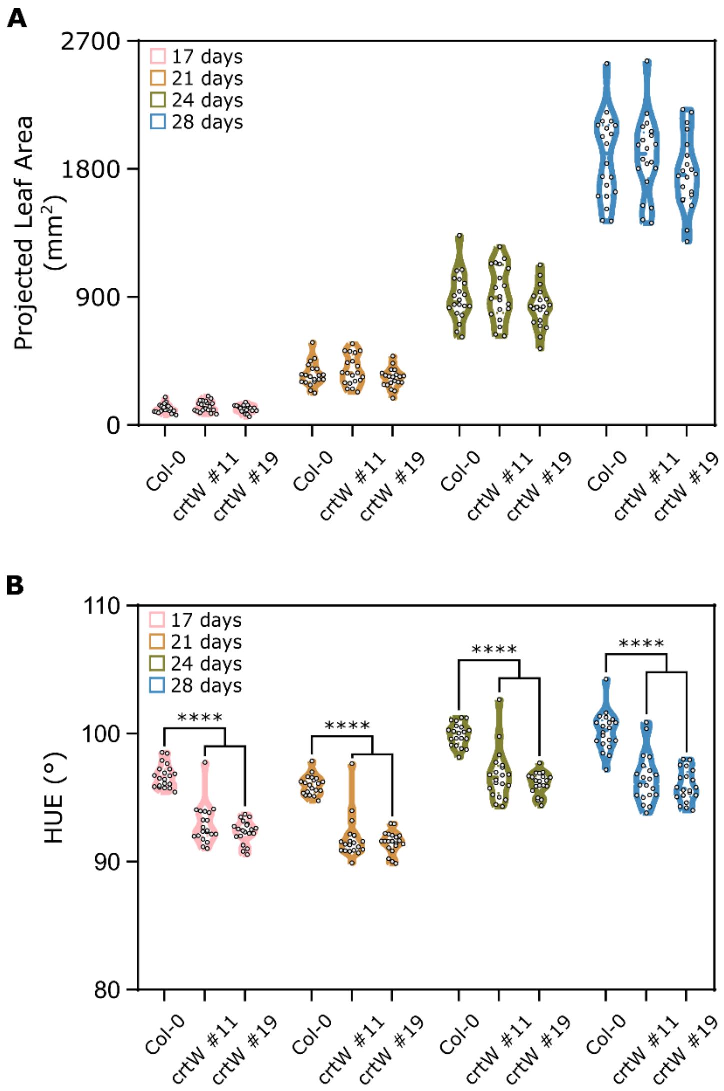

Supplemental Figure S2. Phenotypic comparison between Col-0 and keto-carotenoid producing plants.

A, Comparison in plant size, calculated as the Projected Leaf Area (PLA) and, B, comparison in the HUE-value between Col-0, *crtW*#11 and *crtW*#19. The analysis was made on 20 replicates, and repeated at different time points: 17, 21, 24 and 28 days after germination. Asterisks indicate statistical differences calculated from one-way ANOVA followed by Tukey's post-hoc test (****, p≤ 0.0001).

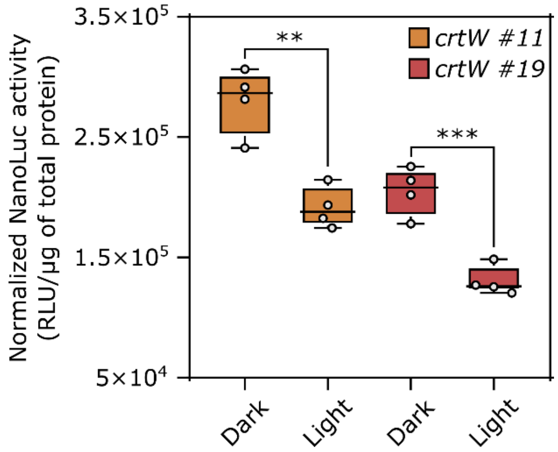

Supplemental Figure S3. Comparison of split-NlucOCP2 activity in two different *crtW* lines.

Light response in Arabidopsis *crtW*#11 and *crtW*#19 isolated protoplasts. Protoplasts were treated with either dark or blue light (350 µmol $µm^{-2}$ s^{-1}) for 20 min. In the box plots, dots represent biological replicates, the black line marks the median, and the box indicates the interquartile range (IQR). Whiskers extend to data points below 1.5 X IQR away from the box extremities. A statistic Student t-test was used to assess significant differences. Asterisks indicate statistical differences (***, p≤ 0.001, **, 0.001<p≤0.01, n=4).

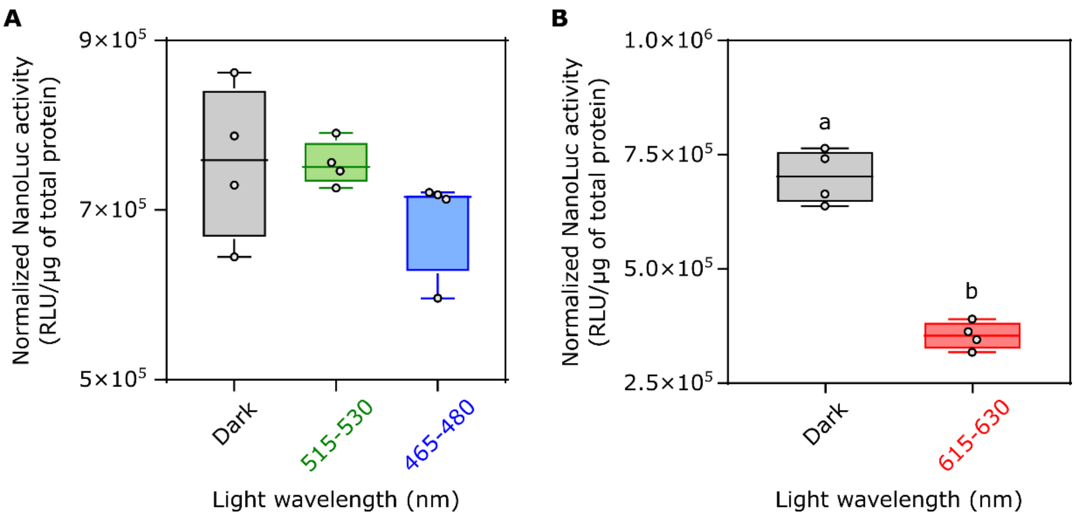

Supplemental Figure S4. Effect of different light spectra on the activity of a synthetic NanoLuc.

The activity of a synthetic Nanoluc was assessed in *crtW*#11 isolated protoplasts under different light regimes. Samples were treated with 350 µmol µm^{-2} s^{-1} light for 20 min. A, Green and blue light treated protoplasts compared with dark treated samples. B, Comparison between dark and red light treated samples. In the box plots, dots represent biological replicates, the black line marks the median, and the box indicates the interquartile range (IQR). Whiskers extend to data points below 1.5 X IQR away from the box extremities. A statistic Student t-test was used to assess significant differences ($p \leq 0.05$, n=4) and they were indicated marked with different letters, when present.

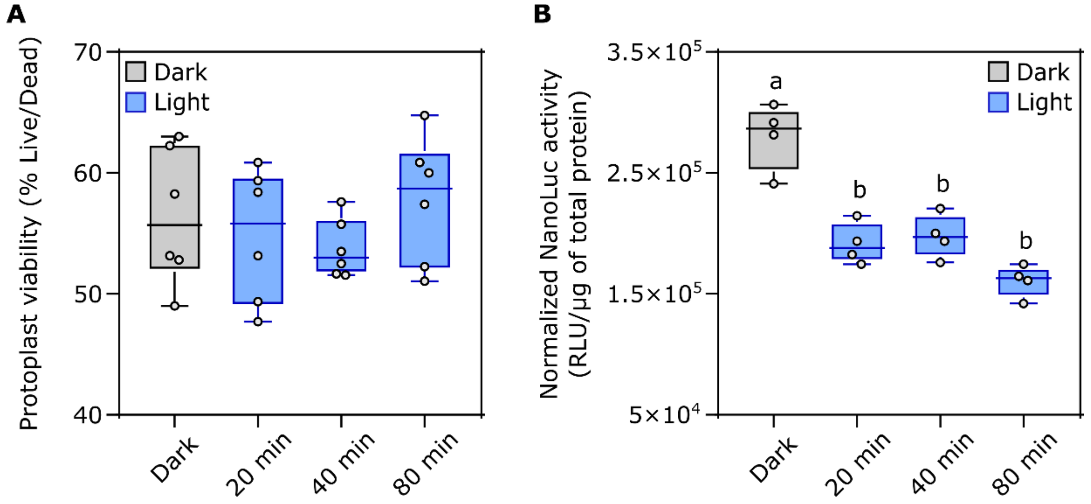

Supplemental Figure S5. Comparison between protoplast vitality and Nanoluc activity in light treated samples.

Protoplasts were treated with either dark or blue light (350 µmol μm^{-2} s^{-1}) for 20, 40 and 80 min. A, Protoplast viability, expressed as % Live/Dead, in dark and light treated samples. B, Nanoluc activity measured in the samples used for vitality check. In the box plots, dots represent biological replicates, the black line marks the median, and the box indicates the interquartile range (IQR). Whiskers extend to data points below 1.5 X IQR away from the box extremities. Different letters indicate statistical differences ($P \leq 0.05$) calculated from one-way ANOVA (n=6) followed by Tukey's post-hoc test.

```
Sig2      RSLDQKIGMNQNLKPSEVIADPEAVTSEDILIKEFMRQDLDKVLDSLGTREKQVIRWRFG      521
RpoD      ISMETPIGDDEDSHLGDFIEDTTLELPLDSATTESLRAATHDVLAGLTAREAKVLRMRFG      564
           *::  ** ::: : .:.* *       *   .* :*   ..** .* :** :*:* ***

Sig2      MEDGRMKTLQEIGEMMGVSRERVRQIESSAFRKLKNKKRNNHLQQYLVAQS      572
RpoD      IDMNTDYTLEEVGKQFDVTRERIRQIEAKALRKLRHPSRSEVLRSFLDD--      613
          :: .   **:*:*: :.*:***:****:.*:***:: .*.: *:.:*
```

Supplemental Figure S6. Multialignment of the 4.3 region (shaded in grey) of *A. thaliana* Sig2 and E. coli RpoD.

RpoD residues involved in AsiA contact are shown in red. Asterisks below a residue indicate identity between the two sequences; ‘:’ and ‘.’indicate similar physical-chemical properties of residues.

Supplemental Table S1. Comparison of the carotenoid content in *crtW* and wild-type Arabidopsis plants.

Data are percentages of each pigment relative to the total carotenoid content per each line. Data are reported as average of three independent replicates. Errors are reported as standard deviation, shown per each group of lines. A Student T-test was used to assess significant differences between *crtW* and Col-0 genotypes. Different letters between rows represent differences in carotenoid content ($p < 0.05$).

Genotype	Neoxanthin	Violaxanthin	Lutein	β-carotene	Astaxanthin	Canthaxanthin
Col-0	14.0% ± 0.3% ^{a}	14.0% ± 0.3% ^{a}	14.0% ± 0.3% ^{a}	14.0% ± 0.3% ^{a}	n.d.	n.d.
crtW11	14.0% ± 0.3% ^{b}	5.3% ± 1.7% ^{b}	49.0% ± 1.2% ^{b}	14.0% ± 0.3% ^{a}	12.5% ± 6.6% ^{b}	1.1% ± 0.6% ^{b}
crtW19	6.6% ± 0.6% ^{c}	3.6% ± 0.6% ^{b}	44.8% ± 2.0% ^{c}	14.0% ± 0.3% ^{a}	23.6% ± 5.2% ^{b}	1.8% ± 0.3% ^{b}

Supplemental Dataset S1. Nucleotide sequences of the constructs used in this work

>PLS-crtW

atggcctcgatgatctcttcttctgctgtgaccactgtgagcagagcttctactgttcagtctgctgctgttgctccttt
cggaggactcaagtctatgactggattcccggtgaagaaggtgaacaccgacatcaccagcatcacctctaacggtggaa
gagtgaagtgttctgctcacgctttgcctaaggctgatctcactgctactagcctcatcgtgagcggaggaattattgct
gcttggctcgctctccatgtgcacgctttgtggtttcttgatgctgctgctcatccgatcctcgctatcgctaatttcct
tggacttacctggctcagcgtgggccttttcattatcgctcatgatgccatgcacggatctgtggttccaggtagaccta
gagctaatgctgctatgggacagcttgttctctggctctacgctggattctcttggcgtaagatgatcgtgaagcacatg
gctcatcacagacacgctggaaccgatgatgatcctgatttcgatcatggtggaccggtgagatggtacgctagattcat
cggaacttacttcggatggcgtgagggacttcttctccctgttatcgttactgtgtacgctctcatcctcggtgaccgtt
ggatgtatgttgtgttctggcctcttccgagcatcctcgcttctatccagcttttcgttttcggaacttggctccctcat
aggcctggacatgatgctttccctgataggcacaacgctcgttcctctaggatctctgatcctgtgtctctcctcacttg
cttccacttcggaggataccaccatgagcatcatctccatcctactgtgccttggtggcgtttgccttctactaggacta
agggtgataccgcctga

>PLS-NTD-linker-NNluc

atggcctcgatgatctcttcttctgctgtgaccactgtgagcagagcttctactgttcagtctgctgctgttgctccttt
cggaggactcaagtctatgactggattcccggtgaagaaggtgaacaccgacatcaccagcatcacctctaacggtggaa
gagtgaagtgctccttcaccatcgagtctgccagatctatcttcccggaaaccgaagtggctaacgctatccctgctatc
gtcgagtctttcaaccagctctctgctgaggatcagcttgctcttttgtggttcgcttacaccgagatgggagtgacaat
taccccctgctgctatgggagctgctaacatggttttcgctgaaaagacgctcacccagatcaagcagatgtctgctagag
aacagacccaggtgatgtgcgatctcatcaaccatgctgatacccctatctgcaggacctacagctctttcggaactaac
gtgaagctcggcttctggtatcaactcggagaatggatgaagcagggaatcgtggctcctattcctgagggatacaagct
ttctgctcaggcttctgatgtgctccagactatcagacagcttgaaggtggacagcagctcactgttctccagaacatcg
tggtgaacatgggatacgctccaggatcttctggtggtggtggaagcggaggtggtggatcatctggtgttttcactctc
gaggacttcgttggagattgggagcaaactgctgcttacaacctcgatcaggttctcgagcaaggtggtgtttcttcact
ccttcagaacctcgctgtgtctgtgactcctatccagaggatcgttaggtctggtgagaacgctctcaagatcgacatcc
acgtgatcatcccttacgagggactttctgctgatcagatggctcagatcgaagaggtgttcaaggttgtgtaccctgtg
gatgaccaccacttcaaggtgatccttccatacggaactctcgtgatcgatggtgtgacaccgaacatgctcaactactt
cggtagaccgtacgagggaatcgctgtgttcgatggaaagaagattaccgtcactgggaccctctggaacgggaacaaga
ttatcgatgagaggctcatcaccccggacggctctatgcttttcagagtgaccatcaacagctga

>PLS-CNluc-linker-CTD

atggcctcgatgatctcttcttctgctgtgaccactgtgagcagagcttctactgttcagtctgctgctgttgctccttt
cggaggactcaagtctatgactggattcccggtgaagaaggtgaacaccgacatcaccagcatcacctctaacggtggaa
gagttaagtgcgtgaccgggtacagactcttcgaggaaatccttggatctagcggaggtggtggatctggtggtggtgga
agttctggaattgagggaatcgataacctcaccgtgctgagctacatggaaaacatgaacgccttcgacttccagggtgc
tgtggctcttttgctgaaaacggtgctctccaacctccgttccaagatcctatcgttgggcaagagaacatcctcgctt
acatgagggaagagtgctacggacttaagctcatccctgagagaggaatctctgagcctgctgaaggcggattcactcag
gttaaggttaccggaaaggtgcagactccatggttcggagattctgtgggaatcaacctcgcttggcgattccttttgaa
cccgcagggaaagatcttcttcgtggctatcgatgtgctcgctacccctcaagagcttctcaacatgggattcatccagt
ga

>NTD—linker-NNluc

atgtccttcaccatcgagtctgccagatctatcttcccggaaaccgaagtggctaacgctatccctgctatcgtcgagtc
tttcaaccagctctctgctgaggatcagcttgctcttttgtggttcgcttacaccgagatgggagtgacaattacccctg
ctgctatgggagctgctaacatggttttcgctgaaaagacgctcacccagatcaagcagatgtctgctagagaacagacc
caggtgatgtgcgatctcatcaaccatgctgataccctatctgcaggacctacagctctttcggaactaacgtgaagct
cggcttctggtatcaactcggagaatggatgaagcagggaatcgtggctcctattcctgagggatacaagctttctgctc
aggcttctgatgtgctccagactatcagacagcttgaaggtggacagcagctcactgttctccagaacatcgtggtgaac
atgggatacgctccaggatcttctggtggtggtggaagcggaggtggtggatcatctggtgttttcactctcgaggactt
cgttggagattgggagcaaactgctgcttacaacctcgatcaggttctcgagcaaggtggtgtttcttcactccttcaga
acctcgctgtgtctgtgactcctatccagaggatcgttaggtctggtgagaacgctctcaagatcgacatccacgtgatc
atcccttacgagggactttctgctgatcagatggctcagatcgaagaggtgttcaaggttgtgtaccctgtggatgacca
ccacttcaaggtgatccttccatacggaactctcgtgatcgatggtgtgacaccgaacatgctcaactacttcggtagac
cgtacgagggaatcgctgtgttcgatggaaagaagattaccgtcactgggaccctctggaacgggaacaagattatcgat
gagaggctcatcaccccggacggctctatgcttttcagagtgaccatcaacagctga

>CNluc-linker-CTD

atggtgaccgggtacagactcttcgaggaaatccttggatctagcggaggtggtggatctggtggtggtggaagttctgg
aattgagggaatcgataacctcaccgtgctgagctacatggaaaacatgaacgccttcgacttccagggtgctgtggctc
ttttgctgaaaacggtgctctccaacctccgttccaagatcctatcgttgggcaagagaacatcctcgcttacatgagg
gaagagtgctacggacttaagctcatccctgagagaggaatctctgagcctgctgaaggcggattcactcaggttaaggt
taccggaaaggtgcagactccatggttcggagattctgtgggaatcaacctcgcttggcgattccttttgaacccgcagg
gaaagatcttcttcgtggctatcgatgtgctcgctacccctcaagagcttctcaacatgggattcatccagtga

>PLS-NNLuc-linker-CNLuc

atggcctcgatgatctcttcttctgctgtgaccactgtgagcagagcttctactgttcagtctgctgctgttgctccttt
cggaggactcaagtctatgactggattcccggtgaagaaggtgaacaccgacatcaccagcatcacctctaacggtggaa
gagtgaagtgcgtgttcaccctcgaagatttcgttggagattgggagcagaccgctgcttacaaccttgatcaggttttg
gagcaaggcggggtttcatctttgcttcagaacctcgctgtgagcgttacccctatccagagaatcgttaggtctggtga
gaacgccctcaagatcgatatccacgtgatcatcccttacgagggactttctgctgatcagatggctcagatcgaagagg
tgttcaaggtggtgtaccctgtggatgaccaccacttcaaggtgatccttccatacggaactctcgtgatcgatggtgtg
actccgaacatgctcaactacttcggtagaccgtacgagggaatcgctgtgttcgatggaaagaagattaccgtcactgg
gaccctctggaacgggaacaagattatcgatgagaggctcatcaccccggacggctctatgcttttcagagtgactatca
acagcgggtcctctggtggtggtggaagcggaggtggtggatcttctggtgttactggataccgactcttcgaggaaatc
ctctga

>PLS-NTD-AsiA

Atggcctcgatgatctcttcttctgctgtgaccactgtgagcagagcttctactgttcagtctgctgctgttgctccttt
cggaggactcaagtctatgactggattcccggtgaagaaggtgaacaccgacatcaccagcatcacctctaacggtggaa
gagtgaagtgctccttcaccatcgagtctgccagatctatcttcccggaaaccgaagtggctaacgctatccctgctatc
gtcgagtctttcaaccagctctctgctgaggatcagcttgctcttttgtggttcgcttacaccgagatgggagtgacaat
taccccctgctgctatgggagctgctaacatggttttcgctgaaaagacgctcacccagatcaagcagatgtctgctagag
aacagacccaggtgatgtgcgatctcatcaaccatgctgatacccctatctgcaggacctacagctctttcggaactaac
gtgaagctcggcttctggtatcaactcggagaatggatgaagcagggaatcgtggctcctattcctgagggatacaagct

ttctgctcaggcttctgatgtgctccagactatcagacagcttgaaggtggacagcagctcactgttctccagaacatcg
tggtgaacatgggatacgctccaggatcttctggtggtggtggaagcggaggtggtggatcatctggtaataagaatatt
gatactgttagagaaattattactgttgcttctattcttattaagttttctagagaagatattgttgaaaatagagctaa
ttttattgcttttcttaatgaaattggagttactcatgaaggaagaaagcttaatcaaaattcttttagaaagattgttt
ctgaacttactcaagaagataagaagactcttattgatgaatttaatgaaggatttgaaggagtttatagatatcttgaa
atgtatactaataagtga

>Sig2-linker-CTD

atgtcttcttgtcttcttcctcagttcaagtgcccacctgattctttctctattcacttccgaacctctttctgtgcccc
taaacacaacaagggttcagtcttcttccaaccgcaatgtgcagtatccacttcaccggcgttattaacttctatgcttg
atgtcgcaaagcttagactaccctctttcgatactgattcggattcccttatatcagacaggcagtggacttatacaagg
cccgatggtccttccactgaggcgaagtatttagaagctttagcctctgagacacttctcacaagcgatgaagcagtagt
tgtagcagcagcagctgaagcagtcgcccttgcaagagctgctgtcaaagttgccaaagatgcaacattatttaagaaca
gtaacaacacgaacctattaacttcgtcaacggccgacaaacgctccaagtgggaccagtttactgagaaggaacgtgct
ggcatattggggcatctagcggtttcggacaatggaattgtgagtgataaaatcactgcatctgcctctaacaaagagtc
tattggtgatttagaatcagaaaaacaagaagaagttgagcttctggaggagcaaccttcagtgagtttagctgtgagat
ctacacgtcaaactgaaaggaaagctcggagggcaaaagggttagagaaaactgcatcaggtattccgtctgtgaagact
ggttcgagccctaaaaagaaacgtcttgttgcgcaggaagttgatcataatgatcctttgcgttatctaagaatgacaac
aagcagttccaagcttctcactgtcagagaagaacatgagctgtcggcaggaatacaggaccttctgaagttagaaagac
ttcaaacagagcttacagagcgtagtggacgtcagccaacctttgcgcagtgggcttctgctgctggagtcgatcagaaa
tcattaaggcaacgtatacatcatggcacactatgcaaagacaaaatgatcaaaagcaacattcgactcgttatttcgat
tgcaaagaattatcaaggagctgggatgaacctccaagatcttgtccaggaagggtgcagagggcttgtgaggggagcag
agaagtttgatgctacaaagggttttaaattttcgacttacgcgcattggtggatcaagcaagctgtgcggaagtctctc
tctgatcagtccagaatgataagattgccttttcacatggtggaagcaacatatagggtgaaagaggcacgaaagcaact
gtacagtgaaaccggtaagcacccaaagaacgaagaaattgcagaggcaacagggctgtcgatgaagagactcatggcgg
ttctactctctcctaaacctccgaggtcgctagaccagaaaatcggaatgaatcaaaacctcaaaccttcggaagtgata
gcagatccagaagcagtaacgtcagaagatatactgataaaggaattcatgaggcaggacttggacaaagtgttggactc
gttgggtacaagggagaaacaagtgatacgttggagatttgggatggaggatgggagaatgaagacgttgcaagagatag
gagagatgatgggagtgagcagggagagagtaagacagatagagtcatctgcattcaggaaactaaagaacaagaagaga
aacaaccatttgcagcaatacttggttgcacaatcatccggatctagcggaggtggtggatctggtggtggtggaagttc
tggatcaggtattgagggaatcgataacctcaccgtgctgagctacatggaaaacatgaacgccttcgacttccagggtg
ctgtggctctttttgctgaaaacggtgctctccaacctccgttccaagatcctatcgttggcaagagaacatcctcgct
tacatgagggaagagtgctacggacttaagctcatccctgagagaggaatctctgagcctgctgaaggcggattcactca
ggttaaggttaccggaaaggtgcagactccatggttcggagattctgtgggaatcaacctcgcttggcgattccttttga
acccgcagggaaagatcttcttcgtggctatcgatgtgctcgctacccctcaagagcttctcaacatgggattcatccag
tga
